# Expression of Mutated *BRAF*^V595E^ Kinase in Canine Carcinomas—An Immunohistochemical Study

**DOI:** 10.3390/vetsci11110584

**Published:** 2024-11-20

**Authors:** Annika Bartel, Heike Aupperle-Lellbach, Alexandra Kehl, Silvia Weidle, Leonore Aeschlimann, Robert Klopfleisch, Simone de Brot

**Affiliations:** 1 LABOKLIN GmbH & Co. KG, 97688 Bad Kissingen, Germany; bartel@laboklin.com (A.B.); kehl@laboklin.com (A.K.); 2 School of Medicine, Institute of Pathology, Technical University of Munich, Trogerstr. 18, 80333 München, Germany; silvia.weidle@tum.de; 3 Institute of Animal Pathology, COMPATH, University of Bern, 3012 Bern, Switzerland; leonore.kuechler@unibe.ch (L.A.); simone.debrot@unibe.ch (S.d.B.); 4 Institute of Veterinary Pathology, Free University of Berlin, 14195 Berlin, Germany; robert.klopfleisch@fu-berlin.de

**Keywords:** dog, molecular profiling, BRAF, genetic, immunohistochemistry, ddPCR

## Abstract

Alterations of the *BRAF* gene and the resulting changes in the BRAF protein are one example of molecular cancer profiling in humans and dogs. We tested 227 samples of canine carcinomas from different anatomical sites (anal sac (*n* = 23), intestine (*n* = 21), liver (*n* = 21), lungs (*n* = 19), mammary gland (*n* = 20), nasal cavity (*n* = 21), oral epithelium (*n* = 18), ovary (*n* = 20), prostate (*n* = 21), thyroid gland (*n* = 21), urinary bladder (*n* = 22)) with two commercially available primary anti-BRAF^V600E^ antibodies (VE1 Ventana, VE1 Abcam). The immunohistochemical results were confirmed with droplet digital PCR (ddPCR). *BRAF^V595E^*-mutated cases were found in canine prostatic (16/21), urothelial (17/22), and oral squamous cell carcinomas (4/18), while other carcinoma types tested negative. Both antibodies showed consistent results, with intracytoplasmic immunolabeling of tumour cells, making them reliable tools for detecting the *BRAF^V595E^* mutation in canine carcinomas. In conclusion, identifying *BRAF* mutations from biopsy material offers a valuable opportunity to enhance cancer treatment strategies (BRAF inhibitors) in canine urothelial carcinomas, prostatic carcinomas, and oral squamous cell carcinomas.

## Simple Summary

Molecular profiling of cancer is used to examine for specific mutations or proteins and is becoming increasingly important in oncology. An example of such a mutation is the altered *BRAF* gene and the resulting changed BRAF protein, which is already known to play a role in urinary bladder and prostatic carcinomas in dogs. We assumed that the mutant BRAF^V595E^ protein should be considered in other canine carcinomas and tested 227 samples of 11 different carcinoma origins (anal sac (*n* = 23), intestine (*n* = 21), liver (*n* = 21), lungs (*n* = 19), mammary gland (*n* = 20), nasal cavity (*n* = 21), oral epithelium (*n* = 18), ovary (*n* = 20), prostate (*n* = 21), thyroid gland (*n* = 21), urinary bladder (*n* = 22)). We used immunohistochemistry with two different primary antibodies, each binding to the altered BRAF protein, and confirmed our results with droplet digital PCR (ddPCR). Among all the tested canine carcinomas, we found *BRAF*-mutated tumours in the prostate (16/21), the urinary bladder (17/22), and the oral cavity epithelium (4/18), while other carcinomas tested negative. Our findings showed that both antibodies are dependable tools for detecting the *BRAF^V595E^* mutation in canine carcinomas. Detecting *BRAF* mutations is important for applying future therapeutic approaches, including BRAF inhibitors.

## 1. Introduction

At 27%, tumour diseases are the most common cause of death in dogs [1], with a significant proportion of carcinomas such as mammary adenocarcinomas or squamous cell carcinomas [2]. Given the prevalence and impact of these tumours, it is essential to understand their underlying molecular mechanisms to make informed clinical decisions. Profiling cancer at a molecular level has become increasingly important. It is already part of the routine diagnostic in human medicine [3] but is still in its beginning in veterinary medicine [4]. One key aspect of molecular characterisation is identifying specific genetic mutations that can drive cancer growth. Among these genetic mutations, alterations in the *BRAF* gene and the resulting change in the BRAF protein are particularly interesting in humans [5] and dogs [4].

**Figure 1:**
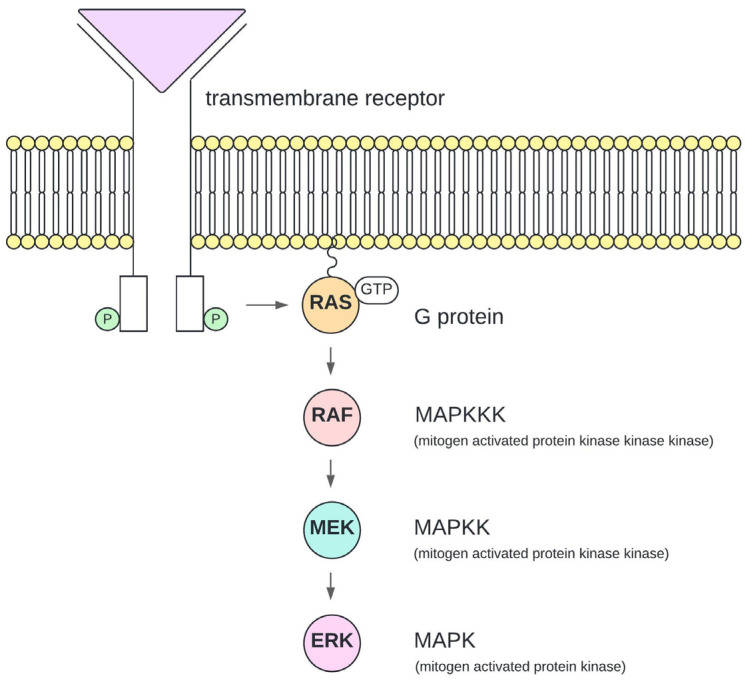
Schematic illustration of the MAPK/ERK pathway (mitogen-activated protein kinase/extracellular signal-regulated kinase pathway). The binding of an extracellular ligand (purple triangle) to a transmembrane receptor leads to the activation of the G-protein RAS (rat sarcoma protein; orange) by replacing GDP with GTP. RAS binds to RAF (rapidly accelerated fibrosarcoma protein; red), leading to the activation of MEK1/2 (MAP/ERK kinase 1/2; blue), which itself activates ERK1/2 (extracellular signal-regulated kinase; pink).

The family of the three RAF proteins (A-RAF, B-RAF, C-RAF) plays a fundamental role in regulating cellular processes in normal mammalian cells, such as proliferation, differentiation, and apoptosis [6]. As serine/threonine kinases, they participate in the mitogen-activated protein kinase/extracellular signal-regulated kinase (MAPK/ERK) pathway (Figure 1). The binding of an extracellular ligand to a transmembrane receptor, such as the EGF receptor, HER2 receptor, or KIT receptor, leads to the activation of the G-protein RAS. Consequently, GTP-bound RAS binds to RAF, activating the mitogen-activated protein kinase kinases MEK1/2, resulting in a phosphorylation of ERK1 and ERK2 [7]. Targets for ERK1/2 are cytosolic and nuclear proteins with wide-ranging effects on the cell, such as the regulation of transcription factors [8]. Somatic activating mutations of RAF increase activation of the MAPK/ERK pathway and promote tumour development [9].

The V600E mutation of *BRAF* is of great importance in human cancer, mostly described in melanomas, papillary thyroid carcinomas, and colorectal carcinomas [10]. The mutant protein presumably mimics the conformational changes after dimerisation, leading to a 500–700-fold increased activity compared to the wild-type BRAF protein [11].

In 2015, Mochizuki et al. hypothesised that *BRAF* gene mutations also exist in canine cancers and sequenced the exon 15 of *BRAF* in 667 canine primary tumours. The results showed that a single nucleotide transversion from thymine to adenine at codon 595 (V595E) occurred in 9.6% of the tested tumours, with a frequency of 80% (20/25) in prostatic carcinoma (PC), 67% (30/45) in urothelial carcinoma (UC), 11% (2/18) in oral squamous cell carcinoma (OSCC), and 6% (1/18) in pulmonary carcinoma [12]. The mutation V595E of canine *BRAF* is congruent with the V600E mutation in human cancer [13].

The high number of *BRAF* mutations in canine PC and UC showed the possible use of the mutation as a molecular marker in diagnostically challenging cancers [14,15]. It is also worth noting that certain Terrier breeds seem to be disposed to *BRAF*-mutated UC [2,16].

Another study used Sanger sequencing and restriction fragment length polymorphism assays to examine canine papillary oral squamous cell carcinoma (pOSCC), finding *BRAF* mutations in 12 out of 14 pOSCC [17].

In human medicine, the detection of *BRAF* mutations in metastatic melanoma patients opened the possibility of BRAF/MAPK-targeted therapy with selective BRAF inhibitors (BRAFi), for instance, vemurafenib or dabrafenib [18]. Similarly, the identification of the orthologous mutation *BRAF* V595E in dogs enabled the possible use of BRAF/MAPK targeted treatments for various canine cancers, especially the specific BRAFi vemurafenib [19] and the non-specific BRAFi sorafenib [20].

The unique possibility to search selectively for the mutated human BRAF^V600E^ protein by immunohistochemistry using a mutation-specific antibody allows a large number of samples to be examined without PCR procedure with all its disadvantages, such as high time and cost expenditure or variable quality of DNA [21]. If present, mutated BRAF is expressed in the cytoplasm of the tumour cell. It is, however, noteworthy that non-specific nuclear staining has been reported with anti-BRAF antibodies from various manufacturers in several studies with human tissue [22,23].

A recent study by Aeschlimann et al. successfully used the anti-human BRAF V600E-specific antibody from Ventana^®^ (Roche Diagnostics) to detect the canine BRAF V595E mutant protein in bladder and prostate tumours in dogs through immunohistochemistry. Results showed evident cytoplasmatic staining in *BRAF*-mutated cancer cells [24]. Another functioning BRAF V600E-specific antibody in human medicine is from the manufacturer Abcam^®^. Although no studies have been published about its efficacy in canine tissue to date, it is worth noting that the manufacturer’s website features an image of a canine *BRAF*-mutated urothelial carcinoma stained with their antibody [25].

Currently, there is deficient literature dealing with the BRAF kinase and its mutations in canine carcinoma other than UC and PC. We hypothesised that mutated BRAF^V595E^ kinase could be relevant for various canine carcinomas of other sites. For these purposes, we evaluated 227 samples of canine carcinomas at 11 different anatomical sites. Furthermore, we compared the results of two primary antibodies used in human medicine for their usability in canine samples and double-checked our results with droplet digital PCR (ddPCR).

## 2. Materials and Methods

### 2.1. Samples

A total of 227 samples from dogs with different types of carcinomas were available as formalin-fixed paraffin-embedded (FFPE) tissue blocks. Included were: 23 anal sac adenocarcinomas, 21 intestinal adenocarcinomas (15 small intestine, 6 large intestine), 21 liver carcinomas (3 cholangiocarcinomas, 18 hepatocellular carcinomas), 20 mammary adenocarcinomas with lymph node metastases, 21 nasal adenocarcinomas, 18 oral squamous cell carcinomas (OSCC), 20 ovarian adenocarcinomas, 21 prostatic carcinomas (PC; 14 adenocarcinomas, 7 prostatic urethral carcinomas), 19 pulmonary adenocarcinomas, 21 thyroid adenocarcinomas, and 22 urothelial carcinomas of the bladder (UC). Included breeds were 67 mongrels, 40 Terriers, 9 Cocker Spaniels, 8 Beagles, 8 Poodles, 5 Australian Shepherds, 5 Dachshunds, and 85 others.

An overview of included cases is provided in Table 1. For detailed information on each case, including the case number and dog’s breed, please refer to Supplementary Table S1.

Samples were provided randomly from Laboklin GmbH and CO.KG out of archived cases from 2017–2024. As all samples were routine diagnostic submissions, there was no need to submit a request for animal testing or to obtain approval from the ethics committee. This was confirmed by the decision of the local government (RUF-55.2.2-2532-1-86-5).

### 2.2. Histology and Tissue Microarray (TMA)

Haematoxylin and eosin (HE)-stained slides were available for each sample.

General inclusion criteria were:

- Unambiguous diagnosis: A precise diagnosis of a carcinoma was possible.
- Typical growth patterns: The carcinoma showed typical growth patterns, categorised based on the classification from *Tumors in Domestic Animals* by Meuten et al. [26]. Carcinomas of the prostate were classified according to the system used by Palmieri et al. [27].
- Minimal inflammatory cells: The carcinoma showed not more than mild infiltration of inflammatory cells.

Tissue microarray (TMA) blocks were created for all 11 tumour types with core diameters of 1.5 mm and multiple cores (2–9) for each tumour. Whenever adjacent normal epithelial tissue of the same origin was available, it was also included in the TMA block. All TMA blocks were manufactured using the TMA Grand Master (3DHistech, Budapest, Hungary) at the Comparative Experimental Pathology department of the Institute of Pathology at the Technical University of Munich in Germany. The regions of interest from each sample were marked on the glass slide and later transferred to the donor blocks for the TMA. Such regions of interest [28] were, for example, different growth patterns within a tumour to portray heterogeneity and non-neoplastic tissue for an internal negative control later on.

### 2.3. Immunohistochemistry

Immunohistochemistry was performed on the slides cut from the TMA blocks. Mouse monoclonal BRAF-V600E antibodies from two different manufacturers (Table 2) were used. Immunohistochemistry was conducted at the University of Bern.

The staining protocol for the Ventana^®^-antibody (Roche Diagnostics, Basel, Swiss) started with preparing the SuperFrost slides (Langenbrinck, Emmendingen, Germany) with ULTRA cell conditioning solution 1 for 72 min at 99 °C, followed by a blocking step with a peroxidase inhibitor (containing 3.0% hydrogen peroxide solution). It was then incubated for 60 min at 36 °C with the primary mouse anti-human antibody targeting the mutated BRAF V600E protein. Detection was carried out using the OptiView DAB IHC Detection Kit (Ventana Medical Systems, Roche, Basel, Swiss) according to the instructions.

For immunohistochemistry with the Abcam^®^-antibody, the SuperFrost slides (Langenbrinck, Emmendingen, Germany) were pretreated and stained on Bond-III immunostainer (Leica Biosystems, Melbourne, Australia). Slides were pretreated with the Bond epitope retrieval solution 2 (pH 9) for 40 min at 100 °C. To reduce the non-specific binding of primary antibodies, a protein block solution (Leica Novocastra, Nussloch, Germany) was applied for 10 min at room temperature for all the following steps. Then, the slides were incubated with the primary anti-BRAF-V600E antibody with the dilution 1:50 for 15 min. All further steps were performed using reagents of the Bond Polymer Refine Detection Kit (Leica Biosystems, Nussloch, Germany) as follows: Endogenous peroxidase was blocked for 5 min, then a secondary antibody was applied (8 min), followed by a peroxidase-labelled polymer (8 min). Both reagents were supplemented with 2% dog serum to block non-specific binding (LabForce, Nunningen, Switzerland). Finally, slides were developed in 3,3′-diaminobenzidine/H_2_O_2_ (10 min), counterstained with haematoxylin, and mounted.

Non-neoplastic tissue was used as an internal negative control for all entities, while BRAF-positive canine UC served as an external on-slide positive control. All TMA slides were stained in the same run. Simultaneously with the immunohistochemical staining, slides from all 11 TMAs were prepared without the primary antibody for negative reagent control (NRC; [30]).

Immunohistochemical findings were examined using light microscopy. The reaction result was assessed as positive if immunolabelling, indicated by brown colour, was localised within the cytoplasm of all or a proportion of tumour cells in at least one TMA core and could be differentiated from any non-specific labelling seen in the NRC or within the sample. Negative results showed no specific binding of the primary antibody. Samples were defined as positive or negative without further subdivisions according to intensity or percentage of positive cells.

### 2.4. Droplet Digital PCR (ddPCR)

Subsequently, each sample from carcinoma types showing positive immunostaining was further investigated with ddPCR for the *BRAF^V595E^* mutation at LABOKLIN GmbH and Co. KG. Sections of the whole tissue block containing the total tumour sample were prepared. Types of carcinomas that were negative in the BRAF-immunohistochemistry for all samples were further investigated by performing a ddPCR from their TMA block.

As starting material, 2 µm sections were prepared according to the manual instructions, and excess paraffin was trimmed off using a scalpel. DNA isolation was performed using the QIAamp^®^ DNA FFPE Tissue Kit (Qiagen, Hilden, Germany) according to the enclosed handbook, followed by the Zymo Research DNA Clean and Concentrator^®^ Kit (Zymo Research Europe GmbH, Freiburg, Germany). Isolated DNA was analysed for the *BRAF* mutation c.1784 T > A by ddPCR using a mutation-specific TaqMan^®^ assay, following the protocol described by Mochizuki et al. [31]. The evaluation used the DropletReader (Bio-Rad, Feldkirchen, Germany) and the QuantaSoft™ Software (Bio-Rad, Feldkirchen, Germany).

## 3. Results

### 3.1. BRAF V595E Immunohistochemistry

Both applied antibodies showed comparable immunolabelling. Positive cases were characterised by the tumour cells’ homogeneous, finely granular, cytoplasmic brown staining. There was mildly varying staining intensity, but a clear distinction from the adjacent non-neoplastic tissue was obvious. Both antibodies directly visualised the mutant protein in the tumour tissue at single-cell resolution and showed valid and concurring results. No intranuclear staining was observed in any of the samples for both antibodies. Non-specific staining could be seen in the same locations for both antibodies, for example, in the keratin pearls of the oral squamous cell carcinoma, leukocytes, especially mast cells, or in the secretions of the mammary glands, the prostate gland, or the intestine.

**Figure 2:**
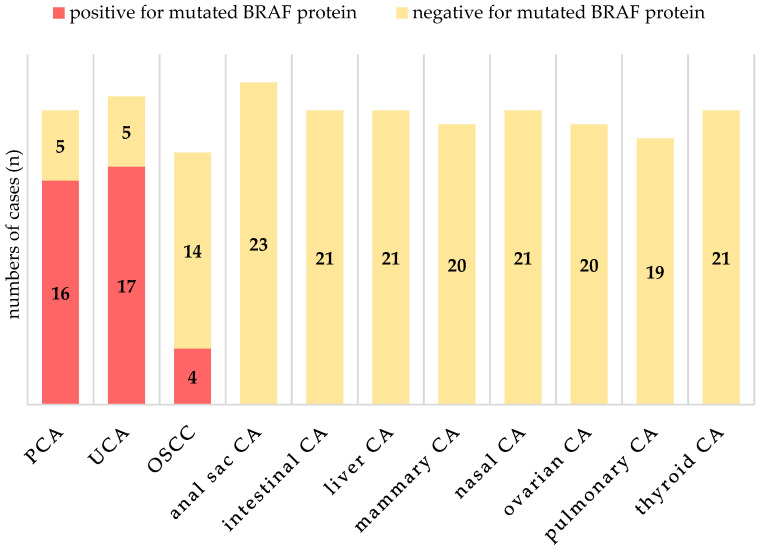
Results of immunohistochemical anti-BRAF^V595E^ staining for all tested entities. Positive cases were found in urothelial carcinomas (UCA), prostatic carcinomas (PCA), and oral squamous cell carcinomas (OSCC).

The majority of positive cases were found in prostatic carcinomas (16/21) and urothelial carcinomas (17/22), with further positive cases in oral squamous cell carcinomas (4/18). All tested samples for anal sac carcinomas (*n* = 23), intestinal adenocarcinomas (*n* = 21), liver carcinomas (*n* = 21), mammary adenocarcinomas (*n* = 20), nasal adenocarcinomas (*n* = 21), ovarian adenocarcinomas (*n* = 20), pulmonary adenocarcinomas (*n* = 19), and thyroid adenocarcinomas (*n* = 21) were negative (Figure 2).

**Figure 3:**
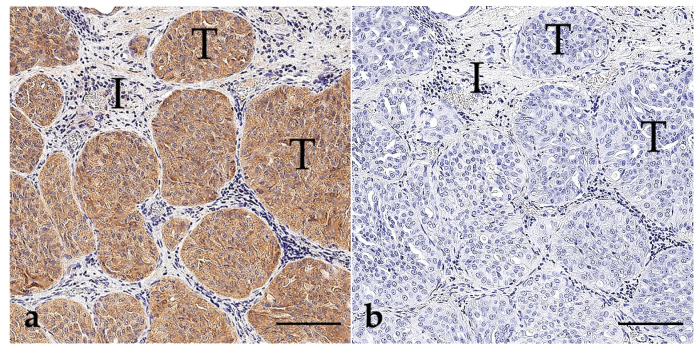

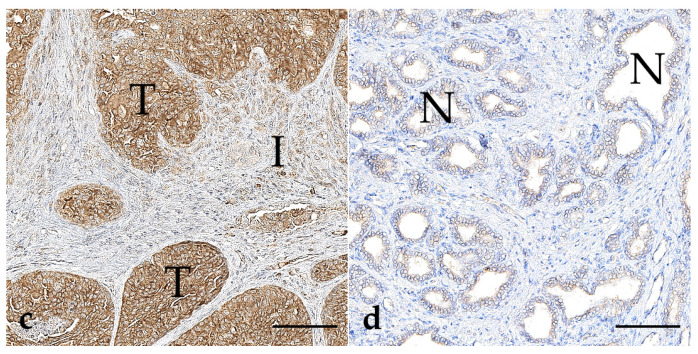
BRAF immunohistochemistry of prostatic carcinomas (**a**–**c**) and normal prostatic tissue (**d**); bar indicates 100 µm for (**a**–**d**). (**a**) Prostatic carcinoma (case 162), stained with the anti-BRAF^V600E^ antibody from Abcam^®^. Tumour cells (T) are intensively labelled, while the non-neoplastic interstitium (I) is negative. (**b**) Negative reagent control to picture (**a**). (**c**) Prostatic carcinoma (case 151), stained with the anti-BRAF^V600E^ antibody from Ventana^®^. Tumour cells (T) show positive immunolabelling, while the interstitium (I) remains negative. (**d**) Non-neoplastic prostatic glands (N) show no labelling, confirming the absence of the mutant BRAF protein.

The positive samples of PCA (Figure 3) showed strong cytoplasmatic immunolabelling within the tumour cells. Out of 16 BRAF-positive prostatic carcinomas, 2 were tubulopapillary adenocarcinomas, 6 were mixed solid-tubular adenocarcinomas, and 8 were prostatic urethral carcinomas. All samples were confirmed with ddPCR with concurring results.

The breeds included in the entire testing group of PC were 7 Terriers, 2 Dachshunds, 2 Poodles, 1 Bulldog, 1 Shi Tzu, 1 Münsterländer, 1 Labrador Retriever, 1 Belgian Shepherd, 1 Boxer, 1 Border Collie, 1 Welsh Corgi, and 2 mongrels. Despite limitations due to low case numbers, a potential association between the dog’s breed and BRAF-mutated carcinomas could be observed. The most frequently affected breed in PCA (*n* = 5) were Terriers.

**Figure 4:**
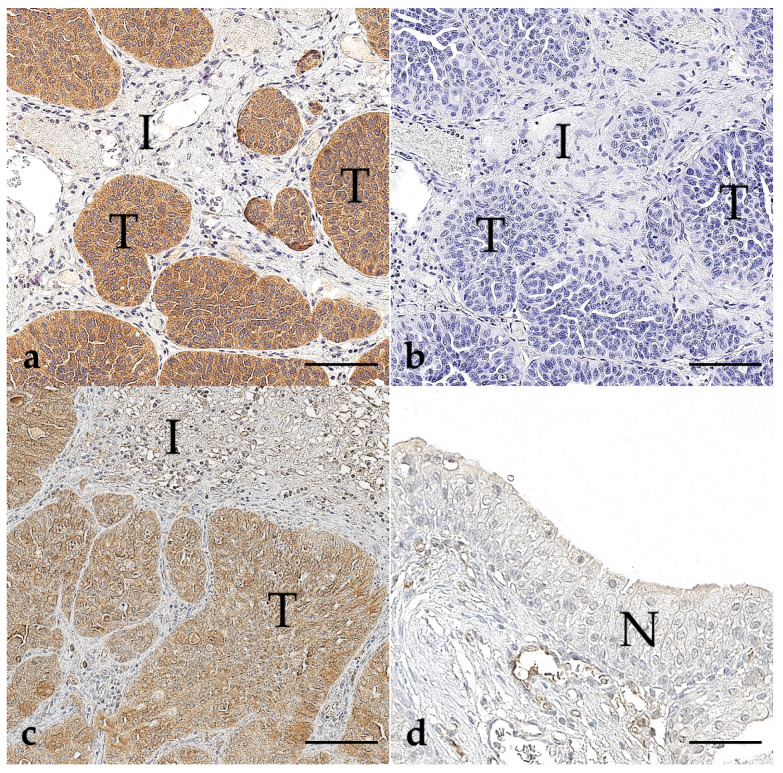
BRAF immunohistochemistry of urothelial carcinomas of the bladder (**a**–**c**) and normal urothelium (**d**); bar indicates 100 µm for (**a**–**c**) and 50 µm for (**d**). (**a**) A typical positive case of UC (case 222), stained with the anti-BRAF^V600E^ antibody from Abcam^®^, showed strong cytoplasmic immunolabeling in tumour cells (T) with a clear distinction from the surrounding tissue (I). (**b**) The corresponding negative reagent control to picture (**a**). (**c**) Another UC case (case 227) with similar results was stained with the anti-BRAF^V600E^ antibody from Ventana^®^. Tumour cells (T) show positive immunolabelling, while the interstitium (I) remains negative. (**d**) Non-neoplastic urothelial epithelium (N) was used as an internal negative control.

Among the 17 positive UCAs (Figure 4), 13 were papillary, 3 were solid, and 1 was a mixed solid-papillary carcinoma. Breeds included were 8 Terriers, 4 Beagles, 3 Border Collies, 1 Nova Scotia Duck Tolling Retriever, 1 Shetland Sheepdog, 1 Samoyede, 1 Pointer, 1 Cocker Spaniel, and 2 Mongrels. As well as for PCA, Terriers were the most frequently affected breed in UCA (*n* = 5).

In one case of UCA (case 220), the immunostaining was weak, but the ddPCR confirmed the presence of the mutation. However, only three positive events were detectable, indicating that the mutation was present in very low quantities.

**Figure 5:**
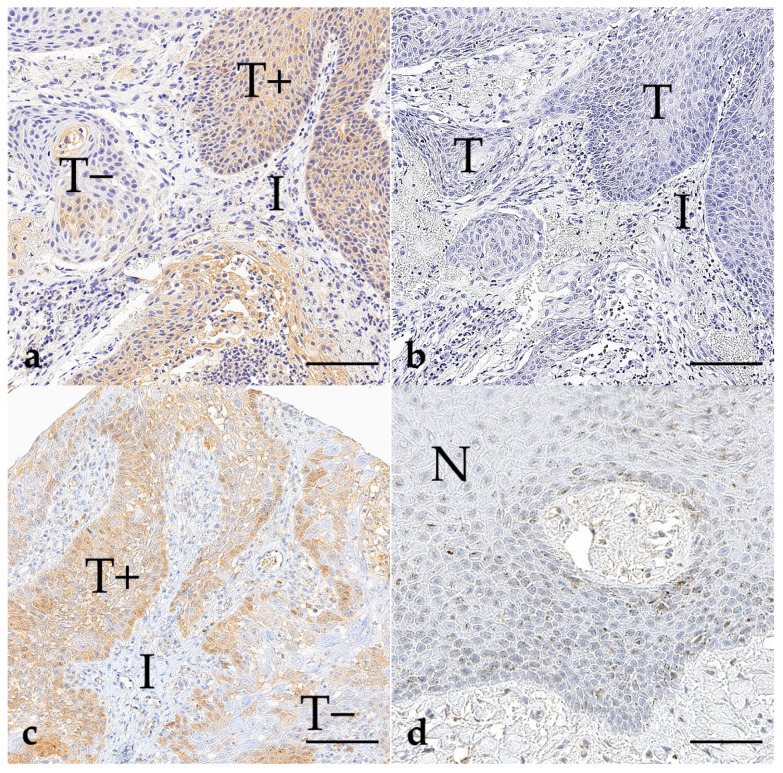
BRAF immunohistochemistry of oral squamous cell carcinomas (**a**–**c**) and normal epithelium (**d**); bar indicates 100 µm for (**a**–**c**) and 50 µm for (**d**). (**a**) An exemplary case of OSCC (case 111), stained with the anti-BRAF^V600E^ antibody from Abcam^®^, shows the intracellular presence of the mutated BRAF protein (T+), while the surrounding interstitial tissue (I) remains unstained. Notably, some parts of the tumour show no immunostaining (T−), highlighting the intratumoral heterogeneity of the BRAF mutation. (**b**) The corresponding negative reagent control to (**a**) confirms the specificity and sensitivity of the primary antibody. (**c**) Another case of OSCC (case 115) demonstrates a partly strong expression of the mutated BRAF protein (T+), as visualised here with the anti-BRAF^V600E^ antibody from Ventana^®^. In contrast, other areas of the tumour show an absence of the mutated BRAF protein (T−). (**d**) Non-neoplastic epithelium (N) (case 115) adjacent to the tumour served as an internal negative control.

The positive samples of oral squamous cell carcinomas (Figure 5) showed varying strong cytoplasmatic immunolabeling within the tumour cells. Compared to TMA-cores of PC and UC, intratumoral heterogeneity of the BRAF mutation was observed in all positive cases. There was unspecific heterogeneous staining of occurring keratin pearls for both antibodies, which could be easily recognised by incorporating the negative reagent control without the primary antibody.

Notably, out of the 4 positive OSCCs, only one showed papillary histomorphology, while the other 3 could be assigned the conventional type. All samples were confirmed by ddPCR with concurring results.

The breeds included were 2 French Bulldogs, 3 Poodles, 1 Terrier, 1 Labrador Retriever, 1 Bearded Collie, 1 German Shepherd, 1 Australian Shepherd, 1 Basset Hound, 1 Magyar Viszla, 1 Bernese Mountain Dog, 3 mongrels, and 2 unknown cases. For OSCC, no association could be observed between the breed of the dog and the occurrence of the mutated BRAF protein.

### 3.2. Droplet Digital PCR (ddPCR)

**Figure 6:**
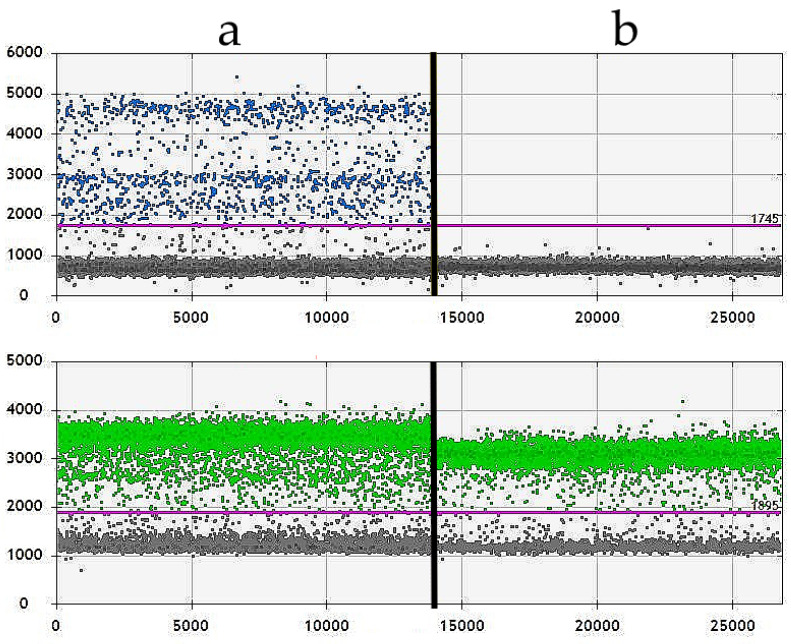
Results from two samples (**a**,**b**) were analysed with ddPCR. Blue dots indicate positive droplets for the *BRAF^V595E^* mutation, while green dots represent wild-type *BRAF*. Grey dots signify droplets that tested negative for both. The violet horizontal line marks the threshold to differentiate positive from negative droplets. (**a**) Results of a conventional OSCC testing positive for the *BRAF^V595E^* mutation (case 115). (**b**) Results of a conventional OSCC testing negative for the *BRAF^V595E^* mutation (case 119).

All samples from UC, PC, and OSCC were further confirmed individually to be *BRAF* positive or negative with consistent results (Figure 6). Out of the 36 cases with positive IHC and one case with mild IHC staining intensity, all 37 were tested positive by ddPCR.

As expected, varying levels of wild-type *BRAF* were confirmed to be present in both *BRAF*-mutated and non-mutated tumours, as shown by ddPCR. Given the negative IHC staining of non-mutated tumours, IHC for BRAF^V595E^ is confirmed to be specific for the mutant protein and does not cross-react with wild-type BRAF.

All types of carcinomas that were negative in the BRAF immunohistochemistry were further investigated by performing a ddPCR from their TMA block as a pool sample. All TMA blocks from anal sac carcinomas, intestinal adenocarcinomas, liver carcinomas, mammary adenocarcinomas, nasal adenocarcinomas, ovarian adenocarcinomas, pulmonary adenocarcinomas, and thyroid adenocarcinomas were negative for the *BRAF^V595E^* mutation in the ddPCR, confirming the immunohistochemical results.

## 4. Discussion

In our study, we confirmed the results of previous studies showing that the *BRAF^V595E^* mutation occurs with high frequency in urothelial carcinomas and prostatic carcinomas, with Terriers being a commonly affected breed [14,24]. Furthermore, we identified the mutation in oral squamous cell carcinomas. In contrast to the study from Peralta et al., who reported *BRAF* mutations exclusively in papillary oral squamous cell carcinomas [17], our findings reveal the presence of *BRAF* mutations in both papillary and non-papillary oral squamous cell carcinoma. Mochizuki et al. also identified *BRAF* mutations in 2 out of 18 oral squamous cell carcinoma samples, although the specific histomorphological subtypes were not reported [12]. It is also worth noting that all three studies, including our own, which examined OSCC, had relatively small sample sizes: Peralta et al. with 24 samples, Mochizuki et al. with 18 samples, and our study with 18 samples. Thus, further studies evaluating the characteristics of OSCC with *BRAF* mutation are necessary.

Although Mochizuki et al. identified *BRAF* mutations in 6% (1/18) of pulmonary carcinomas they examined [12], none of our samples (*n* = 19) showed positive staining in IHC or tested positive in ddPCR, suggesting a low prevalence of *BRAF^V595E^* mutation in canine pulmonary carcinomas. It is important to mention that, unlike the study by Mochizuki et al., where the specific growth patterns remain unknown, we differentiated the tested carcinomas according to their growth patterns. Our tested samples for pulmonary carcinomas showed 11 tubulopapillary, 7 mixed solid and tubular, and one lepidic growth.

Notably, in human medicine, *BRAF* mutations are recognised for their diagnostic and predictive significance in thyroid carcinomas [32], pulmonary carcinomas [33], colorectal carcinomas [34], and hepatocellular carcinomas [35], while in contrast, our tested samples in these specific cancer types tested negative for the *BRAF* mutation. On the other hand, carcinomas of the prostate and bladder with frequent *BRAF^V595E^* mutations in dogs rarely have the concurring *BRAF^V600E^* mutation in people [36], indicating that *BRAF* mutations are specific for certain types of carcinoma and differ between humans and dogs.

To briefly address feline oncology, it is important to emphasise that the genetics of feline tumours have not been studied as extensively as those in dogs. However, to our knowledge, the *BRAF* mutation located at exon 15 appears relatively uncommon in cats [37].

In our study, we demonstrated that the anti-BRAF^V600E^ antibody from both manufacturers Ventana^®^ and Abcam^®^ is a reliable and valid method for detecting the mutant BRAF^V595E^ protein in canine carcinomas, underlining the results from Aeschlimann et al. [24]. Despite seeing non-specific immunolabeling in various locations, such as mast cells or glandular secretion, the assessment of tissue as either positive or negative for BRAF^V595E^ mutated protein was clear and straightforward for all examined tumour types. However, this highlights the importance of performing a negative control without the primary antibody. It is worth mentioning that there appears to be heterogeneity in the number of mutated cells in urothelial carcinomas [24] and during neoplastic transformation. The mild immunohistochemical labelling in one UCA case was reflected by the minimal number of mutated cells detected by ddPCR. Thus, in routine diagnostics, uncertain cases in IHC should be confirmed by additional ddPCR. On the other hand, a case report from a dog with a non-invasive nodular lesion in the bladder mucosa showed a BRAF^V595E^ mutation, indicating early detection of the mutation in dysplastic tissue [38].

Although nonspecific intranuclear staining was seen in previous studies in human medicine [22,23], it could not be detected in our samples.

In summary, using BRAF^V600E^-specific antibodies to evaluate canine tissues offers a fast and cost-efficient method for assessing BRAF status in FFPE tissue. Furthermore, heterogeneity within the tumour can only be observed through IHC, while ddPCR doesn’t capture this variability. However, it is important to note that various preanalytical factors, such as fixation time, temperature, and tissue processing methods, can affect the quality of IHC results in FFPE tissues [39].

In the context of translational medicine, which aims to bridge basic science and clinical practice [40], implementing BRAF IHC into routine diagnostics is essential for veterinary pathology. Currently, in veterinary medicine, the detection of *BRAF* mutations is primarily used for diagnostic purposes rather than therapeutic strategies. Detecting *BRAF* mutations from urine using ddPCR [41] offers a valuable option if the histological or cytological diagnosis of transitional cell carcinoma remains unclear and can even be used as a screening test in predisposed breeds, such as Terriers.

Currently, limited treatment options are available for various canine cancers, such as prostatic carcinomas [42]. Thus, the identification of *BRAF* mutations from FFPE biopsy material could provide a valuable opportunity to enhance routine cancer treatment strategies not only in human medicine but in veterinary oncology as well. In human medicine, BRAF inhibitors have shown significant clinical benefits for patients with *BRAF^V600E^* mutated cancer and have been approved by the United States Food and Drug Administration (FDA) [43]. Chapman et al. showed in 2011 that treatment with the selective BRAFi vemurafenib improved survival rates in patients with *BRAF^V600E^* mutated melanoma. The overall survival of 675 patients after 6 months was 84% compared to 64% treated with dacarbazine, the previous gold standard treatment [18]. In the meantime, several studies in human medicine have shown promising results for the therapy with vemurafenib for tumours other than melanoma, such as hairy cell leukaemia [44] and pancreatic ductal adenocarcinomas [45]. Nevertheless, it must be noted that the appearance of a *BRAF* mutation does not always correlate with the clinical response to BRAF inhibitors. Resistance to BRAFi through reactivation of the MAPK/ERK pathway has been described for various reasons [46]. To ensure the effectiveness of BRAF inhibitors, despite the possibility of several resistance mechanisms, the combination with a MEK and/or EGFR inhibitor has improved performance in human patients compared to BRAFi monotherapy [47].

To assess the significance of the canine *BRAF^V595^* mutation, BRAFi have been tested in dogs [19] with BRAF-mutated urothelial carcinoma and several canine cell lines [48]. Rossmann et al. performed a clinical trial with vemurafenib in 34 dogs with naturally occurring invasive urothelial carcinoma harbouring the *BRAF* mutation, showing a significant initial response to the treatment in 9 out of 24 dogs, followed by drug resistance. Those findings reflect the challenges faced in human cancer treatments with BRAFi [19]. A case report from Kim et al. evaluated the antitumor effect of sorafenib on a metastatic urethral transitional cell carcinoma (TCC) in a 10-year-old dog. They simultaneously monitored the treatment response by assessing the circulation of cell-free tumour DNA. A reduction in measured mutation levels was associated with a favourable therapeutic response, while an increase indicated disease progression [20]. Furthermore, sorafenib seems to be well tolerated in dogs [49]. In 2021, Jung et al. established novel canine TCC cell lines from two tumour tissues and one metastatic lymph node from canine patients with *BRAF*-mutated TCC and compared the effect of sorafenib and vemurafenib on these three canine cell lines and one human *BRAF*-mutated TCC cell line. The results demonstrated that the canine cell lines were more sensitive to sorafenib than vemurafenib [48].

To expand the possibility of therapeutic approaches, the detection of *BRAF* mutations represents one important method for cancer diagnostics, in addition to traditional diagnostic methods. Nevertheless, it is essential to highlight that the patient’s signalment and histomorphological characteristics of each tumour must also be considered in addition to molecular characterisation. Comprehensive interpretation is needed to guide treatment decisions and individualise medicine.

In conclusion, this study demonstrated that the expression of mutated BRAF kinase is present in canine prostatic carcinomas, urothelial carcinomas, and oral squamous cell carcinomas, highlighting the need for further evaluation, particularly in squamous cell carcinomas. The *BRAF^V595E^* mutation seems to be neglected in the other tested carcinomas. However, it is important to note that the sample sizes investigated in the literature and our study were relatively small overall. We showed that the anti-BRAF^V600E^ antibodies used in human medicine from Ventana^®^ and Abcam^®^ are valid options for diagnosing BRAF^V595E^ mutations of different canine carcinoma origins. As soon as the use of BRAFi becomes more relevant as a therapeutic option, the possibility of performing immunohistochemistry with reliable antibodies is of great importance for clinicians and pathologists, especially if there is no easy access to PCR.

## Figures and Tables

**Table 1:** Overview of included cases examined for the mutated BRAF^V595E^ protein.

Origin of Carcinoma	Age Range (Years)	Sex	Growth Patterns
Anal sac (*n* = 23)	5–12	4 f, 3 fn, 5 m, 11 mn	13 solid, 3 tubular, 7 mixed solid-tubular
Intestine (*n* = 21; 15 small intestine, 6 large intestine)	7–14	9 f, 2 fn, 8 m, 2 mn	15 tubular, 4 mucinous, 1 solid, 1 mixed solid-tubular
Liver (*n* = 21; 18 hepatocellular; 3 cholangiocarcinoma)	7–16	5 f, 10 fn, 5 m, 1 mn	9 solid, 7 trabecular, 2 clear-cell, 2 tubulopapillary, 1 mixed solid-tubular
Lungs (*n* = 19)	6–13	5 f, 4 fn, 4 m, 6 mn	11 tubulopapillary, 7 mixed solid-tubular, 1 lepidic
Mammary gland (*n* = 20)	4–13	10 f, 10 fn	11 tubulopapillary, 4 solid, 5 anaplastic, additional lymph node metastases for each case
Nasal cavity (*n* = 21)	3–13	6 f, 3 fn, 8 m, 4 mn	9 tubular, 7 solid, 5 mixed solid-tubular
Oral epithelium (*n* = 18)	5–14	7 f, 7 fn, 1 m, 3 mn	16 conventional, 2 papillary
Ovary (*n* = 20)	3–14	13 f, 7 fn	17 tubulopapillary, 2 solid, 1 mixed solid-tubular
Prostate (*n* = 21)	7–13	5 m, 16 mn	7 mixed solid-tubular, 4 tubulopapillary, 1 acinar, 9 prostatic urothelial
Thyroid gland (*n* = 21)	5–15	7 f, 10 fn, 2 m, 2 mn	16 mixed follicular and solid, 5 follicular
Urinary bladder (*n* = 22)	7–14	6 f, 9 fn, 4 m, 3 mn	15 papillary, 6 solid, 1 mixed solid-papillary

Abbreviations: f, female; fn, female neutered; m, male; mn, male neutered.

**Table 2:** Overview of both used anti-BRAF V600E antibodies [25,29].

Primary Antibody	Anti-BRAF V600E	Anti-BRAF V600E
Clone	VE1 Mouse Monoclonal Antibody	VE1 Mouse Monoclonal Antibody
Manufacturer	Ventana^®^ (Roche Diagnostics, material number: 08033706001, catalogue number: 760–5095)	Abcam^®^ (ref. ab228461)
Isotype	IgG_2a_	IgG_2b_
Dilution	Diluted in buffer (pH 7.3) with carrier protein Brij 35 and the preservative 0.05% Pro Clin 300	Diluted in buffer (pH 7.5) with carrier protein Tris and the preservative 0.1% Sodium azide

## Data Availability

The original contributions presented in the study are included in the article/Supplementary Materials, further inquiries can be directed to the corresponding author/s.

## Supplementary Materials

The following supporting information can be downloaded at https://www.mdpi.com/article/10.3390/vetsci11110584/s1, Table S1: Information for all included dogs with type of tumour, sex, breed, age at the time of sampling and histological type.
