# Supplementary material for: Expression of Mutated BRAFV595E Kinase in Canine Carcinomas—An Immunohistochemical Study
Source: Vet Sci. 2024 Nov 20;11(11):584. doi: 10.3390/vetsci11110584 (PMC11598906; doi:10.3390/vetsci11110584)
Supplement: Supplementary file 1 [file vetsci-11-00584-s001.zip › vetsci-3307804-supplementary.pdf]

Table S1. Information for all included dogs with type of tumour, sex, breed, age at the time of sampling and histological type. Samples tested positive for the mutated $BRAF^{V595}$ kinase are highlighted in green.

Type of tumour (number of samples)	Sex (neutering status if known)	Breed	Age at the time of sampling in years	histological type, intravascular tumour cell detection if present
**Anal sac carcinoma (23)**				
1	female	crossbreed	7	solid with partly rosette-like formation
2	male (neutered)	Cavalier King Charles Spaniel	10	solid, intravascular tumour cell detection
3	male	Dachshund, Long Haired	10	tubular with partly rosette-like formation
4	male (neutered)	Cocker Spaniel	10	mixed solid and tubular
5	male	Golden Retriver	8	solid
6	female	crossbreed	10	mixed solid and tubular
7	male	Pinscher	10	tubular with partly rosette-like formation
8	female	Dobermann	5	tubular
9	male	German Shepherd	10	solid, intravascular tumour cell detection
10	female (neutered)	crossbreed	9	solid
11	female (neutered)	crossbreed	10	mixed solid and tubular
12	male (neutered)	crossbreed	10	solid with partly rosette-like formation, intravascular tumour cell detection
13	male (neutered)	Rhodesian Ridgeback	7	mixed solid and tubular, intravascular tumour cell detection
14	male (neutered)	crossbreed	10	solid
15	male (neutered)	Australian Shepherd	12	solid with partly rosette-like formation
16	male (neutered)	crossbreed	9	solid, intravascular tumour cell detection
17	female (neutered)	Cocker Spaniel	8	solid with partly rosette-like formation, intravascular tumour cell detection
18	female	Briard	10	mixed solid and tubular
19	male (neutered)	crossbreed	12	solid, intravascular tumour cell detection
20	male (neutered)	German Shepherd	7	mixed solid and tubular
21	male (neutered)	Labrador Retriever	11	solid, intravascular tumour cell detection
22	male (neutered)	crossbreed	12	mixed solid and tubular, intravascular tumour cell detection
23	male	Mountain Dog, Appenzeller	11	solid with partly rosette-like formation
**intestinal adenocarcinoma (21)**				
a) small intestine (15)				
24	female	Terrier, West Highland White	unknown	tubular with partly mucinous differentiation
25	female	crossbreed	10	tubular, intravascular tumour cell detection
26	male	Retriever, Flat Coated	10	solid
27	female	Terrier, West Highland White	13	mucinous
28	female (neutered)	unknown	unknown	tubular with partly mucinous differentiation
29	female	Pinscher, Miniature	7	mucinous
30	male	crossbreed	10	mucinous, intravascular tumour cell detection
31	male (neutered)	Schnauzer, Miniature	9	tubular with partly mucinous differentiation
32	female (neutered)	Pointer, German Shorthaired	11	tubulopapillary
33	male	Terrier, Jack Russel	12	papillary
34	male	Shih Tzu	9	papillary
35	female	Lapphund, Finish	11	tubulopapillary
36	female	crossbreed	unknown	tubular
37	female	crossbreed	14	tubulopapillary
38	female	Pekingese	unknown	tubulopapillary
b) large intestine (6)				
39	male	Terrier, Yorkshire	8	tubular
40	male	Border Collie	14	tubular with partly mucinous differentiation
41	female	Australian Shepherd	10	tubular
42	male (neutered)	Terrier, West Highland White	10	tubular
43	male	Chihuahua	8	mucinous
44	male	crossbreed	9	mixed solid and tubular
**liver carcinoma (21)**				
a) cholangiocarcinoma (3)				
45	female (neutered)	Bobtail	10	tubulopapillary with partly pappilliform differentiation
46	male	Border Collie	16	mixed solid and tubular
47	female (neutered)	crossbreed	12	tubular
b) hepatocellular carinoma (18)				
48	female (neutered)	Dachshund	12	solid
49	female (neutered)	crossbreed	9	trabecular with partly solid formations
50	male (neutered)	Terrier, Jack Russel	13	clear-cell-type with partly solid formations
51	male	Terrier, Yorkshire	9	trabecular
52	male	Terrier, Parson Russel	13	solid
53	female	crossbreed	11	trabecular
54	female (neutered)	Terrier, Scottish	10	trabecular
55	female	crossbreed	10	solid
56	female (neutered)	Terrier, Welsh	14	clear-cell-type with partly solid formations
57	male	Terrier, Irish	10	solid
58	male	Terrier, Yorkshire	11	trabecular with partly solid formations
59	female (neutered)	Dachshund, Wire Haired	10	solid
60	female (neutered)	crossbreed	10	solid
61	female (neutered)	crossbreed	12	solid
62	female (neutered)	crossbreed	9	trabecular
63	female	Bearded Collie	11	solid
64	female	Cocker Spaniel	13	solid
65	female	Poodle	7	trabecular with partly solid formations
**mammary adenocarcinoma (20)**				
66	female (neutered)	Terrier, West Highland	11	tubulopapillary with lymph node metastasis
67	female (neutered)	crossbreed	11	anaplastic with lymph node metastasis
68	female	crossbreed	11	tubulopapillary with lymph node metastasis
69	female (neutered)	Pinscher	12	tubulopapillary with lymph node metastasis
70	female	Dobermann	9	anaplastic with lymph node metastasis
71	female (neutered)	Cocker Spaniel	12	tubulopapillary with lymph node metastasis
72	female	crossbreed	11	solid with lymph node metastasis
73	female	German Shepherd	4	solid with lymph node metastasis
74	female (neutered)	crossbreed	8	anaplastic with lymph node metastasis
75	female (neutered)	Bull Terrier, Miniature	11	solid with lymph node metastasis
76	female	Maltese dog	8	tubulopapillary with lymph node metastasis
77	female	Münsterländer, Small	8	tubulopapillary with lymph node metastasis
78	female (neutered)	Pointer, Hungarian	13	anaplastic with lymph node metastasis
79	female	Terrier, Yorkshire	13	tubulopapillary with lymph node metastasis
80	female	Poodle	13	tubulopapillary with lymph node metastasis
81	female (neutered)	Boxer	11	anaplastic with lymph node metastasis
82	female (neutered)	unknown	8	solid with lymph node metastasis
83	female	crossbreed	12	tubulopapillary with lymph node metastasis
84	female (neutered)	Cocker Spaniel	10	tubulopapillary with lymph node metastasis
85	female	Terrier, Yorkshire	9	tubulopapillary with lymph node metastasis

**nasal adenocarcinoma (21)**				
86	male	crossbreed	8	mixed solid and tubular
87	male (neutered)	crossbreed	9	solid
88	male	crossbreed	13	tubular
89	female (neutered)	crossbreed	11	solid
90	female (neutered)	Rhodesian Ridgeback	6	mixed solid and tubular
91	male	Terrier, Jack Russel	13	solid
92	female	crossbreed	12	mixed solid and tubular
93	female	crossbreed	11	mixed solid and tubular
94	female	Cocker Spaniel	9	solid
95	male (neutered)	Terrier, Yorkshire	12	tubular
96	male	Mountain Dog, Greater Swiss	3	solid
97	male	crossbreed	11	tubular
98	male	crossbreed	6	solid
99	male	Cavalier King Charles Spaniel	9	tubular
100	female	Golden Retriever	11	tubular
101	female	Beagle	11	tubular
102	male	Cocker Spaniel	13	tubular
103	male (neutered)	crossbreed	10	solid
104	female (neutered)	crossbreed	12	tubular
105	male (neutered)	crossbreed	12	tubular
106	female	unknown	5	mixed solid and tubular
**oral squamous cell carcinoma (18)**				
107	female	Bulldog, French	10	conventional
108	female	Poodle	12	conventional
109	female	Labrador Retriever	5	conventional
110	male (neutered)	Poodle	8	conventional
111	male (neutered)	crossbreed	9	papillary
112	female	unknown	8	conventional
113	female (neutered)	crossbreed	12	conventional
114	female (neutered)	Bearded Collie	12	conventional
115	male	German Shepherd	8	conventional
116	female (neutered)	Basset Hound	11	conventional
117	male (neutered)	Australian Shepherd	8	papillary
118	female (neutered)	Magyar Viszla	14	conventional
119	female	unknown	unknown	conventional
120	female (neutered)	Poodle	11	conventional
121	female	crossbreed	12	conventional
122	female (neutered)	Bulldog, French	9	conventional
123	female (neutered)	Terrier, Jack Russel	13	conventional
124	female	Mountain Dog, Bernese	9	conventional
**ovarian adenocarcinoma (20)**				
125	female (neutered)	Terrier, Yorkshire	10	tubulopapillary
126	female	crossbreed	14	solid, intravascular tumour cell detection
127	female	Malteser	3	tubulopapillary
128	female	Golden Retriever	12	tubulopapillary
129	female	crossbreed	11	tubulopapillary
130	female	crossbreed	13	solid
131	female	crossbreed	12	tubulopapillary
132	female (neutered)	crossbreed	13	tubulopapillary
133	female	Shi Tzu	11	tubulopapillary
134	female	Spitz	14	tubulopapillary
135	female	crossbreed	12	tubulopapillary
136	female	crossbreed	14	mixed solid and tubular
137	female (neutered)	crossbreed	13	tubulopapillary, intravascular tumour cell detection
138	female	Golden Retriever	9	tubulopapillary, intravascular tumour cell detection
139	female (neutered)	Terrier, Yorkshire	8	tubulopapillary
140	female	Golden Retriever	14	tubulopapillary
141	female (neutered)	Pointer, German Shorthaired	8	tubulopapillary, intravascular tumour cell detection
142	female (neutered)	Rough Collie	10	tubulopapillary
143	female	Beagle	11	tubulopapillary
144	female (neutered)	Border Collie	8	tubulopapillary
**prostatic carcinoma (21)**				
a) adenocarcinoma (12)				
145	male	Dachshund	11	tubulopapillary, intravascular tumour cell detection
146	male (neutered)	Bulldog, French	11	tubulopapillary, intravascular tumour cell detection
147	male	Dachshund	11	mixed solid and tubular
148	male (neutered)	Shi Tzu	10	mixed solid and tubular
149	male (neutered)	Poodle	7	mixed solid and tubular, intravascular tumour cell detection
150	male	Bullterrier, Staffordshire	9	tubulopapillary
151	male (neutered)	crossbreed	12	mixed solid and tubular
152	male (neutered)	Poodle	12	mixed solid and tubular
153	male	Münsterländer, Small	8	mixed solid and tubular, intravascular tumour cell detection
154	male (neutered)	Labrador Retriever	10	mixed solid and tubular
155	male (neutered)	Belgian Shepherd	9	acinar
156	male	Boxer	10	tubulopapillary
b) prostatic urethral carcinoma (9)				
157	male (neutered)	crossbreed	13	urothelial
158	male (neutered)	Terrier, Jack Russel	unknown	urothelial
159	male (neutered)	Terrier, Yorkshire	7	urothelial
160	male (neutered)	Terrier, Jack Russel	unknown	urothelial
161	male (neutered)	Welsh Corgi	11	urothelial
162	male (neutered)	Bullterrier, Staffordshire	10	urothelial
163	male (neutered)	Terrier, Jack Russel	12	urothelial
164	male (neutered)	Border Collie	12	urothelial
165	male (neutered)	Bullterrier, Staffordshire	10	urothelial
**pulmonary adenocarcinoma (19)**				
166	male (neutered)	crossbreed	12	tubulopapillary, intravascular tumour cell detection
167	male	Galgos Español	10	lepidic
168	male (neutered)	Poodle	6	tubulopapillary
169	female (neutered)	crossbreed	12	mixed solid and tubular
170	female (neutered)	Podenco Andaluz	unknown	tubulopapillary
171	female (neutered)	crossbreed	10	mixed solid and tubular, intravascular tumour cell detection
172	male	crossbreed	8	tubulopapillary
173	male (neutered)	crossbreed	6	tubulopapillary
174	female (neutered)	Australian Shepherd	10	mixed solid and tubular
175	female	Labrador	7	tubulopapillary, intravascular tumour cell detection
176	male	crossbreed	9	tubulopapillary
177	female	crossbreed	7	mixed solid and tubular
178	female	Dobermann	9	tubulopapillary
179	male (neutered)	Labrador	9	mixed solid and tubular
180	female	Cocker Spaniel	unknown	mixed solid and tubular
181	male (neutered)	Terrier	12	tubulopapillary
182	male (neutered)	Terrier, Jack Russel	13	tubulopapillary
183	female	Podenco Andaluz	7	mixed solid and tubular
184	male	crossbreed	10	tubulopapillary

**thyroid adenocarcinoma (21)**				
185	female	Münsterländer, Small	12	mixed follicular and solid, intravascular tumour cell detection
186	female (neutered)	Terrier, Jack Russel	14	mixed follicular and solid, intravascular tumour cell detection
187	female	Sheepdog, Pyrenean	7	mixed follicular and solid, intravascular tumour cell detection
188	male	Labrador Retriever	7	mixed follicular and solid, intravascular tumour cell detection
189	female (neutered)	Terrier, Jack Russel	11	follicular, intravascular tumour cell detection
190	female (neutered)	Dalmatian dog	12	mixed follicular and solid
191	female (neutered)	crossbreed	6	mixed follicular and solid, intravascular tumour cell detection
192	female	crossbreed	15	mixed follicular and solid
193	female (neutered)	Australian Shepherd	10	follicular
194	female	crossbreed	10	mixed follicular and solid
195	male (neutered)	Golden Retriver	13	mixed follicular and solid, intravascular tumour cell detection
196	female (neutered)	crossbreed	14	mixed follicular and solid, intravascular tumour cell detection
197	female	crossbreed	10	follicular, intravascular tumour cell detection
198	female (neutered)	Boxer	6	mixed follicular and solid, intravascular tumour cell detection
199	male (neutered)	Golden Retriver	9	follicular
200	female (neutered)	Beagle	9	mixed follicular and solid
201	female (neutered)	Azawakh	8	mixed follicular and solid, intravascular tumour cell detection
202	female	Saluki	9	follicular
203	female (neutered)	Beagle	12	mixed follicular and solid
204	female	crossbreed	5	mixed follicular and solid, intravascular tumour cell detection
205	male	crossbreed	12	mixed follicular and solid
**urothelial carcinoma of the bladder (22)**				
206	male (neutered)	Beagle	11	solid
207	female	Terrier, Yorkshire	8	papillary
208	male	Bullterrier, Staffordshire	9	solid
209	female (neutered)	Beagle	12	papillary
210	female	Beagle	10	papillary, intravascular tumour cell detection
211	female	crossbreed	14	papillary
212	female (neutered)	Cocker Spaniel	12	solid
213	female	Border Collie	11	papillary
214	female (neutered)	Terrier, Welsh	12	papillary
215	female (neutered)	Tolling Retriever	11	papillary, intravascular tumour cell detection
216	female (neutered)	Terrier, Scottish	11	solid
217	male	Terrier, Yorkshire	8	papillary
218	female (neutered)	Terrier, Scottish	7	mixed solid and papillary, intravascular tumour cell detection
219	male (neutered)	Terrier, Jack Russel	13	papillary
220	female (neutered)	Border Collie	10	solid
221	male	Beagle	7	solid
222	female	Sheepdog, Shetland	10	papillary
223	female	crossbreed	12	papillary
224	female (neutered)	Samojede	12	papillary
225	female (neutered)	Border Collie	11	papillary
226	male (neutered)	Terrier, West Highland White	9	papillary
227	male	Pointer	11	papillary
**Total 227**				
